# Natural variation in SAR11 marine bacterioplankton genomes inferred from metagenomic data

**DOI:** 10.1186/1745-6150-2-27

**Published:** 2007-11-07

**Authors:** Larry J Wilhelm, H James Tripp, Scott A Givan, Daniel P Smith, Stephen J Giovannoni

**Affiliations:** 1 Department of Microbiology, Oregon State University, Corvallis, OR, 97331, USA; 2 Center for Genome Research and Bioinformatics, Oregon State University, Corvallis, OR, 97331, USA

## Abstract

**Background:**

One objective of metagenomics is to reconstruct information about specific uncultured organisms from fragmentary environmental DNA sequences. We used the genome of an isolate of the marine alphaproteobacterium SAR11 ('*Candidatus *Pelagibacter ubique'; strain HTCC1062), obtained from the cold, productive Oregon coast, as a query sequence to study variation in SAR11 metagenome sequence data from the Sargasso Sea, a warm, oligotrophic ocean gyre.

**Results:**

The average amino acid identity of SAR11 genes encoded by the metagenomic data to the query genome was only 71%, indicating significant evolutionary divergence between the coastal isolates and Sargasso Sea populations. However, an analysis of gene neighbors indicated that SAR11 genes in the Sargasso Sea metagenomic data match the gene order of the HTCC1062 genome in 96% of cases (> 85,000 observations), and that rearrangements are most frequent at predicted operon boundaries. There were no conserved examples of genes with known functions being found in the coastal isolates, but not the Sargasso Sea metagenomic data, or vice versa, suggesting that core regions of these diverse SAR11 genomes are relatively conserved in gene content. However, four hypervariable regions were observed, which may encode properties associated with variation in SAR11 ecotypes. The largest of these, HVR2, is a 48 kb region flanked by the sole 5S and 23S genes in the HTCC1062 genome, and mainly encodes genes that determine cell surface properties. A comparison of two closely related '*Candidatus *Pelagibacter' genomes (HTCC1062 and HTCC1002) revealed a number of "gene indels" in core regions. Most of these were found to be polymorphic in the metagenomic data and showed evidence of purifying selection, suggesting that the same "polymorphic gene indels" are maintained in physically isolated SAR11 populations.

**Conclusion:**

These findings suggest that natural selection has conserved many core features of SAR11 genomes across broad oceanic scales, but significant variation was found associated with four hypervariable genome regions. The data also led to the hypothesis that some gene insertions and deletions might be polymorphisms, similar to allelic polymorphisms.

## Open peer review

Reviewed by Eugene Koonin, Igor B. Jouline (Zhulin) and Peer Bork. For the full reviews, please go to the Reviewers' comments section.

## Background

A particularly vexing aspect of microbial genomics is the common observation of high genome variability among strains of a species [1-3]. Such observations have raised significant questions about the validity of the microbial species concept, and the value of single genome sequences for comparisons between taxa [4]. To reconcile this dilemma, it has been suggested that bacterial species have a "core-genome" consisting of genes that are always present, and a "pan-genome" of genes that are variably present [3]. Metagenomics, the study of genome sequence retrieved from mixed assemblages of organisms collected from nature, is providing high coverage of genome sequence variation from natural microbial communities [5], which can be employed to study the conservation of genome features and illustrate patterns of natural variation.

### The Sargasso Sea metagenomic data

The Sargasso Sea is an oligotrophic subtropical gyre where average surface temperatures are about 23°C, and rarely drop below 20°C [6]. The Sargasso Sea metagenomic data consists of 1.6 G base pairs of unique environmental genomic DNA shotgun sequence. The SAR11 clade accounts for 380 of the 1,412 SSU rRNA genes in the Sargasso Sea data (27%), suggesting that it includes enough SAR11 genome sequence data to encode the equivalent of about 775 SAR11 strain HTCC1062 genomes [7]. Despite the abundance of SAR11 genome sequences in the Sargasso Sea data, the assembly of SAR11 genomes failed when traditional DNA assembly methods were applied [7]. The largest SAR11 rRNA-anchored scaffold reconstructed with the Celera Assembler was relatively small (ca. 21,000 bp), and assembly depth-of-coverage was low (0.94 – 2.2 fold) [7].

### Genome streamlining

The genome streamlining theory was invoked to explain the small genomes of '*Candidatus *Pelagibacter' and *Prochlorococcus *[8,9]. The essence of the genome streamlining theory is that selection is most efficient in microbial populations that have large effective population sizes, and therefore the elimination of unnecessary DNA from genomes will be most pronounced in organisms, like bacterioplankton, that meet this criterion. In particular, "genome streamlining" usually refers to the elimination of functionless DNA from genomes, because of the cost of replication. However, in principle the same concept should apply to other features of genome evolution. If the genome streamlining theory is correct, then, in large bacterioplankton populations, selection should be unusually efficient at preserving all genome features that have a positive fitness associated with them, and eliminating features that confer a negative fitness.

### Ecologically significant variation in SAR11 populations

This observation of high sequence diversity led to speculation that the SAR11 clade might be a diverse assemblage of perhaps hundreds or thousands of species, each with low coverage in the shotgun sequence library [7]. However, ecological data suggests that the SAR11 clade consists of a few ecotypes, which can be differentiated either phylogenetically [10], or by their appearance in the environment at different depths and seasons [11]. A phylogenetic analysis of 16S rRNAs revealed the presence of two SAR11 ecotypes in the Sargasso Sea metagenomic data [12]. Rusch et al. [13] reported evidence of nine genetically divergent SAR11 populations in metagenomic data from different ocean surface sites. The interpretation most consistent with this data is that the large population sizes of SAR11, and the age of these clades, allow them to accumulate very extensive neutral sequence variation that renders assembly difficult, but that there are relatively few, perhaps less than a dozen, ecotypes that are important to the ecology of the oceans [2].

Throughout this paper we refer to genes found in the metagenomic data as "SAR11 genes" if they were found on fragments of DNA sequence that harbored at least one gene identified as a '*Candidatus *Pelagibacter' gene, according to the tests described below. We use the term 'Pelagibacter', to refer to genes from the two sequenced genomes of '*Candidatus *Pelagibacter ubique', because these isolates are genetically distinct from Sargasso Sea SAR11 populations. Pelagibacter strains HTCC1062 and HTCC1002 were isolated from the cold, nutrient rich Oregon coast [14,15], have an average 16S rRNA sequence similarity to SAR11 16S rRNA genes from the Sargasso Sea metagenomic data of 96%, and belong to a 23S-ITS-16S phylogenetic cluster that is distinct from Sargasso Sea populations [16]. In addition to ocean currents and geography, the large temperature difference between the Sargasso Sea and the Oregon coast is most likely a significant barrier that isolates their respective SAR11 populations. A SAR11 strain obtained very recently from the Sargasso Sea, HTCC7211, shows a higher temperature optimum than the Oregon coast isolates, as predicted from the significant difference in temperature between these environments (Stingl, unpublished data).

### Autecology approaches to the study of metagenomic data

Autecology is the study of the ecology of species. A variety of approaches have been employed to infer information about microbial genome variation for specific taxa from metagenomic data, with the intent of understanding the roles of organisms in ocean surface ecology. Hallam et al. [17], used a composite genome sequence of *Cenarchaeum symbiosum *to study the diversity of Marine Group I archaeal genomes in metagenomic databases using BLAST score ratios [18] to identify conserved genes, and a similar approach was employed by Coleman to study genome variation in *Prochlorococcus *[19]. Recently, a study was published by Rusch et al. [13] that used marine microbial genomes as templates to identify environmental fragments from ocean metagenomic data. Rusch et al. [13] used BLASTN to identify homologs to SAR11 genes, but did not employ further tests to exclude homologs originating from non-SAR11 organisms.

### Objectives

The objective of this study was to identify genomic features of SAR11 that are conserved between two Pelagibacter isolates and their counterparts in the Sargasso Sea. The predicted proteins of the HTCC1062 genome were used as TBLASTN queries to identify homologs to SAR11 proteins in the Sargasso Sea metagenomic DNA, and only genes that retrieved Pelagibacter homologs as their top scores when the NCBInr database was searched with BLASTP were classified as SAR11 genes. We quantified gene-to-gene boundaries to assess gene insertions, deletions and rearrangements, and the occurrence of non-orthologous genes adjacent to Pelagibacter orthologs. We measured synteny and displayed the relationship between synteny and amino acid identity in a novel way that allows observation of small-scale gene insertions (<5 genes). Collectively, these tools enabled us to ask questions about the presence of genes in conserved gene order, gene rearrangements, and the juxtaposition of Pelagibacter homologs with genes that are not found in Pelagibacter, which might reveal genes unique to the Sargasso Sea population. To our surprise, with the exception of the hypervariable regions of the genome, we found that the genomes of the divergent population we studied were remarkably similar to the query genome in gene content and gene order. The results suggest that extraordinarily high allelic variation and genome rearrangements mask the conservation of many genome properties in native SAR11 populations.

## Results

The genome sequence of any individual microbial cell is a sub-sample of natural variation. Metagenomics is now providing extraordinary datasets that deeply sample natural variation, raising the question, how should this variation be represented? We have taken a comparative approach to learn more about SAR11 genomes, but we have also searched for evolutionary models that will help us understand and graphically represent natural genome variation. Our comparative approach stems from a larger goal of understanding the nature of species and ecotypes in SAR11 populations. A full description of a species or ecotype might include: 1) a list of core genes that confer a relatively conserved phenotype, 2) a list of ancillary genes that may be found, along with the probabilities that each gene is present, and 3) information about patterns in gene composition and gene order that reflect evolutionary processes, such as the divergence of species or ecotypes.

**Figure 1:**
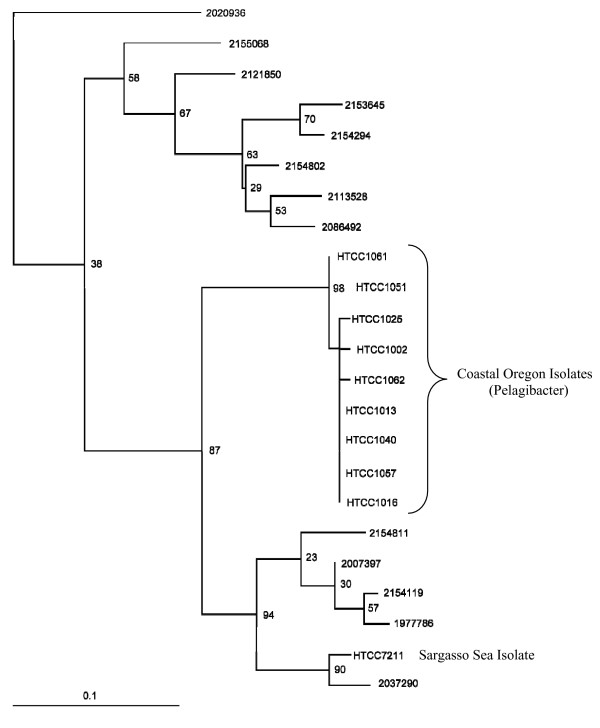
**Maximum likelihood tree of recA amino acid sequences**. The tree includes Sargasso Sea metagenomic data and predicted proteins from cultured isolates of Pelagibacter. The sequence data was derived from Vergin et al., 2007 [37], with the addition of a sequence from strain HTCC7211, which was recently isolated from the Sargasso Sea. All other HTCC strains are from the coastal Pacific Ocean. The non-HTCC numbers correspond to the fragment identifier in the Sargasso Sea data set. The scale bar indicates substitutions per nucleotide position.

Phylogenetic relationships between the query genome and SAR11 genomes represented in the metagenomic data are shown in Fig. 1. The tree supports the presence of two sub-clades in the Sargasso Sea metagenomic data, as previously reported [12].

### Strategy

**Figure 2:**
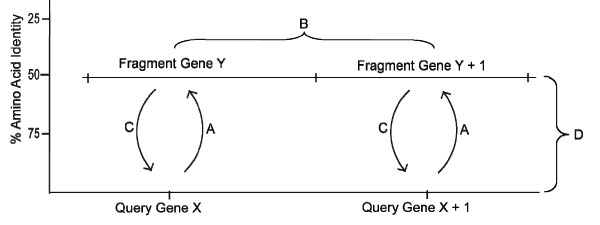
**Schematic diagram of procedures used to "bin" the classes of metagenomic data**. The query genome, in this case HTCC1062, is represented on the x-axis. A) TBLASTN of protein sequences from the query genome against metagenomic data. "Homologous fragments" were defined as fragments of metagenomic data with expect scores of 1 × 10^-10 ^or better to genes from the query genome. B) "Homlogous fragments with synteny" contain homologs in the same gene order as the query genome, with as many as 5 gene gaps (gene deletions) allowed. C) Best-hit test. Fragments of metagenomic data pass the test if the nucleotide sequence of the fragment gene yields the corresponding query gene as the best hit in a BLASTX search of the NCBI nr database. D) The position of the fragment on the vertical axis corresponds to the average amino acid identity score of all the genes on the fragment.

The bioinformatic strategy we employed is outlined in Fig. 2. Table 1 lists the number of fragments in the metagenomic dataset, and the number of those that had TBLASTN expect scores lower than 1 × 10^-10 ^to HTCC1062 genes, which we called "homologous fragments". We use the term "fragment" to refer to DNA sequences from the Sargasso Sea environmental data set, whether they are single reads or contigs assembled from multiple reads. Table 1 also lists the number of homologous fragments that retrieved HTCC1062 or HTCC1002 genes as their best BLAST hits against the NCBInr database. These we called "homologous fragments that passed the best-hit test". We then identified fragments that were syntenic with the query genome, and those that were not. The former we called "syntenic fragments". We used these classes of fragments to study the conservation of amino acid identity, gene content and gene order in the Sargasso Sea SAR11 metagenome.

**Table 1:** Classes ("bins") of metagenomic data fragments used in this study.

**Class**	**Number**	**Genome coverage**	**Avg. AA Identity**
CDSs in HTCC 1062	1354	--	--
Total fragments of metagenomic data	811,372	--	--
Homologous fragments (Fig. 1A)	349,742	97%	--
Homologous fragments that passed the best hit test (Fig. 1C)	187,844	97%	59.7%
Homlogous fragments with synteny	111,332	97%	64.0%
Syntenic fragments* (Fig. 1D)	71,696	91%	71.0%

* For all genes on the fragment, HTCC1062 genes are the best BLAST hits.

**Figure 3:**
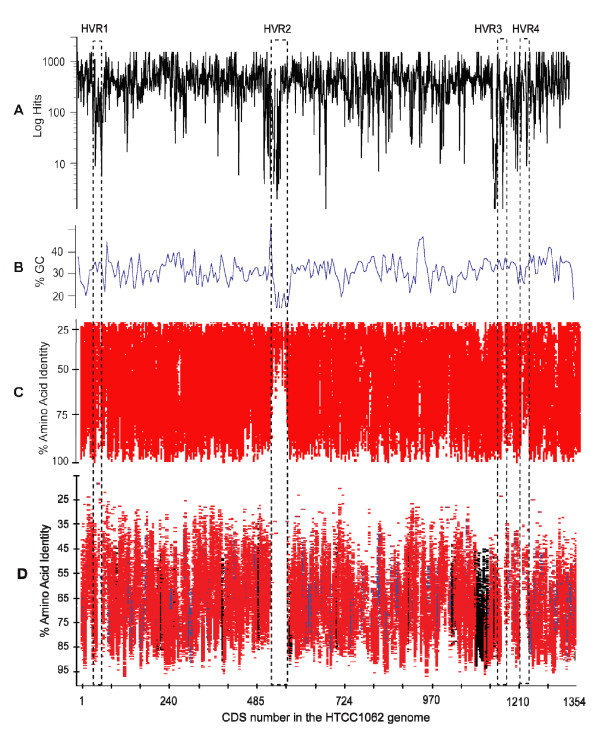
**HTCC1062 genome coverage for the different classes of metagenomic data**. The data for the figure are described numerically in Table 1. A). The number of homologous fragments (TBLASTX expect scores ≤ 1 × 10^-10^) for each HTCC1062 gene, plotted by position in the HTCC1062 genome. B) GC content of HTCC1062 genome. C) The distribution of homologous fragments that passed the best-hit test, regardless of synteny. The data in this plot includes fragments that cover one or more genes. The plotted amino acid identities are for the individual genes, not averaged as they are in the syntenic fragment plot below. D) Syntenic fragment coverage of the HTCC1062 genome as a function of gene position and amino acid identity. See Fig. 2 for an explanation of syntenic fragments. Fragments in this category ("bin"), include parts of at least two genes that could be identified by TBLASTX. Regions of blue on the fragments indicate gaps. Syntenic fragments were allowed to be missing as many as five intervening genes (gaps) between the syntenic genes. Genes that encode ribosomal proteins are indicated in black.

An expect score of 1 × 10^-10 ^was employed in the initial homolog detection step (Fig. 3A). This is a relatively permissive cutoff that ensures the inclusion of homologs, including those from distant taxa, such as other alphaproteobacteria. For convenience, we hereafter refer to this set of fragments as 'homologous fragments'. The log of the number of homologous fragments for each HTCC1062 Coding Sequence (CDS) is shown as a function of gene position in Fig. 3A. We found that homologous fragments from the Sargasso Sea cover 97% of the HTCC1062 genome and account for 43% of the fragments in the complete dataset (349,742 of 811,372, Table 1). This number is somewhat high when compared to predictions from SSU rRNA data – SAR11 genes account for 27% of the total SSU rRNA genes found among the fragments. This observation is not unexpected, considering that the set of homologous fragments contains homologs from non-SAR11 species as well as genes originating from SAR11. Of the 1,354 CDSs in the HTCC1062 genome, 32 are found on at least 3,000 environmental fragments (the maximum number returned from our homolog search), and 31 are not found at all. Fig. 3A reveals three regions of the HTCC1062 genome where coverage is low. These hypervariable regions are labeled HVR1 through HVR4. The longest of these, HVR2, spans almost 50 CDSs.

### Synteny

We define "syntenic fragments" as homologous fragments that passed the best-hit and synteny tests (Fig. 2B,C). Of the 349,742 fragments shown in Fig. 3A, 111,332 share synteny with HTCC1062. A large proportion of the "homologous fragments with synteny" (71,696) passed the best-hit test and were therefore placed in the "syntenic fragments" bin (Table 1, Fig. 3D). Several lines of evidence indicate that the process used to "bin" sequences originating from SAR11 was neither too stringent nor too relaxed. The maximum in the distribution of expect scores for genes on syntenic fragments is 1 × 10^-33 ^and declines sharply as it approaches 1 × 10^-10^, suggesting that the most abundant clade of genes in the dataset, which is predicted to be SAR11, are included, but the tail of the distribution, is excluded (Additional file 1). Also, the average GC content of the syntenic fragments, 29.1%, is nearly identical to that of the HTCC1062 genome (29.7%) and the plotted values approximate a normal distribution (Additional file 2). Tests that used other query genomes (*E. coli*, *Prochlorococcus marinus *and *Croceibacter atlanticus*) supported the conclusion that the procedure used to identify syntenic fragments is selective (Table 2, Additional files 3 and 4).

**Table 2:** Summary of syntenic fragment analysis of 7 marine microbial species and *E. coli*.

Species	CDS	Homologous fragments	Homologous fragments with synteny	Syntenic fragments	% unique to group
'*Candidatus *Pelagibacter ubique'	1,332	349,742	99,457	71,696	98.8
*Prochlorococcus marinus *(MED4)	1,713	226,594	21,304	8,398	99.9
*Croceibacter atlanticus*	2,633	256,645	15,089	1,258	99.0
*Oceanicola batsensis*	4,614	354,793	49,278	532	60.5
*Oceanicaulis alexandrii*	3,365	330,225	36,845	500	31.0
*Escherichia coli*	4,289	306,293	33,306	406	92.9
*Parvularcula bermudensis*	2,824	310,044	30,809	239	31.8
*Janibacter sp*.	4,367	240,360	18,570	23	91.3

Note that "syntenic fragments" are "homologous fragments with synteny" that pass the best hit test. The percent of syntenic fragments which are unique to the query genome, for this set of queries, is shown in the final column.

To visualize variation among the syntenic fragments, the data were plotted as a function of gene position in the HTCC1062 genome and amino acid identity (Fig. 3D). The vertical axis is inverted (low amino acid identity scores at the top) to emphasize the high-scoring syntenic fragment's relationship to the template genome. The syntenic fragments shown in Fig. 3D vary in average amino-acid identity score from 30% to 98%, with an average of 71.0%. The ranges spanned on the vertical axis vary between genes because the amino acid sequences of some genes are more conserved than others. For example, genes for ribosomal proteins appear relatively low in the plots (Fig. 3D, regions colored black). Though 86% of the syntenic fragments carry just two genes, the distribution of syntenic fragments across the HTCC1062 genome is very similar when syntenic fragments carrying three or more genes are plotted (Additional file 5).

The Sargasso Sea metagenome data includes assemblies that are known to contain some errors resulting from unrelated fragments being joined incorrectly [20]. However, an analysis of syntenic fragments that did not include assemblies (Additional file 6) yielded similar plots that supported the same conclusions.

**Figure 4:**
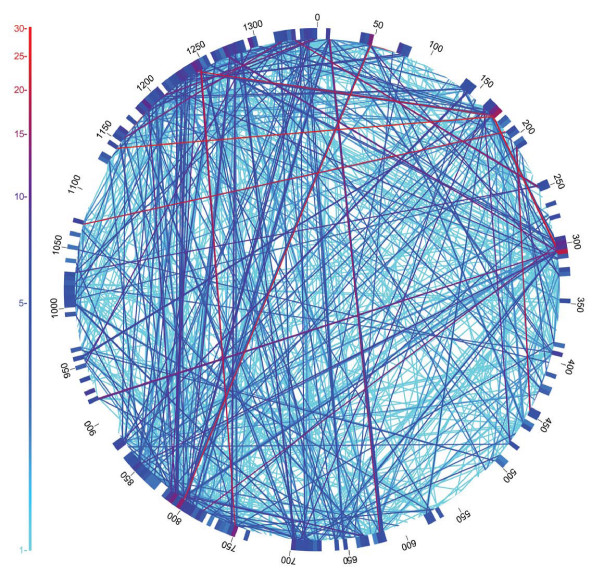
**Re-arrangements in the order of SAR11 genes in the Sargasso Sea metagenome, relative to the HTCC1062 genome**. The genome of HTCC1062 is represented by the outer circle. Internal lines (chords) indicate SAR11 gene rearrangements found on environmental sequence fragments. The number of occurrences of each gene rearrangement is indicated by the color scale.

Syntenic fragments cover most of the HTCC1062 genome (Fig. 3D), indicating that gene order is relatively conserved between the Pacific coastal strains and the Sargasso Sea SAR11 population. An analysis of gene-to-gene boundaries revealed that gene order in 96% of the Sargasso Sea SAR11 homologous fragments matches the gene order of the HTCC1062 genome (see synteny index, in Methods). While the aforementioned facts are striking, they nonetheless allow considerable latitude for genome rearrangements. To map genome rearrangements we plotted the gene positions found on non-syntenic fragments (Fig. 4). These are fragments containing two or more adjacent SAR11 genes that differ from HTCC1062 in gene order. Fragment inclusion in this set follows the same rules as those in Fig. 2, except no synteny requirement was imposed. The high incidence of synteny (Fig. 3D) can be reconciled with the many gene rearrangements shown in Fig. 4 by considering that a few very frequent rearrangements account for most of the cases of rearranged genes. 3,350 fragments had at least two genes that passed the best-hit test but displayed an alternate gene order. Of the 627 different genes found in non-syntenic positions in Fig. 4, seventeen can be tracked to genes that are not found on any syntenic fragments (Table 3). These are likely to represent examples of gene re-arrangements that are conserved in the Sargasso Sea SAR11 population relative to the Oregon coast population.

**Table 3:** Genes from the coastal SAR11 strains with homologs in the Sargasso Sea data, but were never found on syntenic fragments.

Gene number	Gene product	Number of homologs found	Occurrences in alternate gene order
SAR11_0079	hypothetical protein ytoq	66	7
SAR11_0171	rhodanese-related sulfurtransferase (ion tranport)	185	3
SAR11_0312*	unknown (potential sulfotransferase domain)	343	19
SAR11_0393	sam-dependent methyltransferase	117	42
SAR11_0461	unknown	66	4
SAR11_0642	trypsin-like serine protease	354	5
SAR11_0691	unknown	22	4
SAR11_0796	aldehyde dehydrogenase	1200	16
SAR11_0815	carbonic anhydrase	52	29
SAR11_0845	steroid monooxygenase (ion transport)	307	17
SAR11_0852	homoserine dehydrogenase	102	8
SAR11_0959	unknown	21	5
SAR11_1071	gcn5-related n-acetyltransferase	18	5
SAR11_1144*	cyclopropane-fatty-acyl-phospholipid synthase	754	48
SAR11_1227	adp-ribosylglycohydrolase	80	29
SAR11_1248	winged helix dna-binding	160	3
SAR11_1347	Unknown	15	3

* found on just one syntenic fragment, or just a few syntenic fragments with low scores

Genome rearrangements were not random, but were concentrated at boundaries between operons. We compared the evidence for gene re-arrangements to the distribution of predicted operon boundaries [21]. The average number of rearrangements detected per gene boundary was 2.58, but the average at boundaries between operons was 3.21, and the average within operons was 1.94. An analysis of variance indicated that these differences are highly significant (see methods). Perhaps not surprisingly, this finding suggests that selection allows rearrangements between operons more frequently than re-arrangements within operons.

The number of Sargasso Sea fragments in SAR11 syntenic fragment plots declines sharply at sequence similarities above 90%, indicating that the coastal isolates are genetically distinct from their counterparts in the Sargasso Sea. Sequence identity between the syntenic genes and the HTCC1062 genome ranged from high (98%) to low (30%) and averaged 71%. SSU rRNA variation supported a similar conclusion: the average sequence identity of SAR11 16S rRNA fragments from the Sargasso Sea metagenomic data to HTCC1062 is 96.0% ± 2.8% (sd), with a median of 96.9% (n = 379). For comparison, the amino acid identity between *E. coli *and *Salmonella *for GroEL is 98% and among *Burkholderia *species is 75%, whereas the lowest SAR11 GroEL syntenic fragments have an amino acid identity of 77% (avg = 0.87). For RecA the numbers are 96% similarity for *E. coli *and *Salmonella*, 92% within the genus *Burkholderia*, and 63% (avg = 0.81) for the lowest syntenic fragments. Hallam et al. observed a similar average amino acid identity (65%) among 4,000 Sargasso Sea fragments related to *C. symbosium*, and conservation of gene-order as well [17]. Coleman et al. [19] observed a protein sequence identity of 80% among 1,574 genes between two strains of the abundant marine cyanobacterium *Prochlorococcus *that are 99.2% similar at the 16S rRNA locus. Syntenic fragment analysis predicts a very similar average sequence identity in the *Prochlorococcus *metagenome (79%, Additional file 4).

The high sequence divergence observed in the metagenomic data cannot be explained by assuming weak selection. The ratio of synonymous to non-synonymous substitution rates for a selection of 19 genes from the syntenic fragment data ranges from 0.04 to 0.23, indicating purifying selection (Table 4). The implication of this observation is that the divergence of amino acid sequences in the Sargasso Sea SAR11 populations is occurring in proteins that serve important functions.

**Table 4:** Non-synonymous (Ka) and synonymous (Ks) substitution rates, and nucleotide diversity at synonymous (Pi(s)) and non-synonymous (Pi(a)) sites in HTCC1062 genes.

**Gene number**	**Gene product, function**	n*	**Ks**	**Ka**	**Ka/Ks**	**Pi(s)**	**Pi(a)**
SAR11_0162	groEL, chaperonin	18	1.0378 (0.257	0.0524 (0.0284)	0.0504 (0.0268)	0.575	0.038
SAR11_0426	suv3, ATP dependent helic	6	1.4618 (0.241	0.1686 (0.0415)	0.1153 (0.0155)	0.618	0.085
SAR11_0428	thlA, acetyl-coa transferase	6	0.6576 (0.680	0.0933 (0.0966)	0.1419 (0.1419)	0.617	0.284
SAR11_0641	recA, recombinase	15	0.8581 (0.255	0.0604 (0.0277)	0.0704 (0.0223)	0.424	0.036
SAR11_0906	dnaE, DNA polymerase	13	1.5300 (1.279	0.2160 (0.1809)	0.1412 (0.1198)	0.592	0.228
SAR11_1122	rpoC, RNA polymerase	5	1.2869 (0.831	0.0535 (0.0356)	0.0415 (0.0269)	0.625	0.070
SAR11_0078	Epimerase	38	0.6909 (0.309	0.1468 (0.0698)	0.2124 (0.0979)	0.525	0.131
SAR11_0267	CHO transport	14	0.9431 (0.274	0.0992 (0.0323)	0.1052 (0.0204)	0.584	0.088
SAR11_0268	CHO transport	17	0.9077 (0.475	0.1090 (0.0612)	0.1200 (0.0743)	0.545	0.113
SAR11_0273	CHO transport	2	2.3162 (0.000	0.2294 (0.000)	0.0991 (0.000)	0.365	0.110
SAR11_0274	CHO transport	3	0.9938 (0.841	0.1177 (0.1019)	0.1184 (0.0838)	0.415	0.124
SAR11_0655	AA transport	6	0.8693 (0.437	0.0370 (0.0163)	0.0425 (0.0274)	0.497	0.042
SAR11_0660	AA transport	8	0.9990 (0.443	0.0776 (0.0394)	0.0777 (0.0365)	0.513	0.076
SAR11_0677	Unknown	5	1.5142 (0.742	0.1599 (0.0940)	0.1056 (0.0477)	0.531	0.531
SAR11_0764	Conserved hypothetical	5	1.5142 (0.742	0.1599 (0.0940)	0.1056 (0.0477)	0.638	0.142
SAR11_1012	Glycosyl transferase	27	1.4290 (1.279	0.3259 (0.2998)	0.2280 (0.2110)	0.552	0.387
SAR11_1174	Phosphate regulation	29	1.2303 (0.585	0.2008 (0.1037)	0.1632 (0.0850)	0.598	0.180
SAR11_1175	Phosphate regulation	20	0.8141 (0.557	0.1070 (0.1140)	0.1314 (0.0950)	0.484	0.101
SAR11_1288	CHO metabolism	3	1.5345 (0.105	0.1548 (0.0377)	0.1009 (0.0190)	0.568	0.158

* number of sequences tested.

Six highly conserved genes are listed first. The remaining thirteen were sampled from the 22 HTCC1062 genes missing from closely related strain HTCC1002 that show variable syntenic fragment coverage. Standard error is shown in parentheses

**Figure 5:**
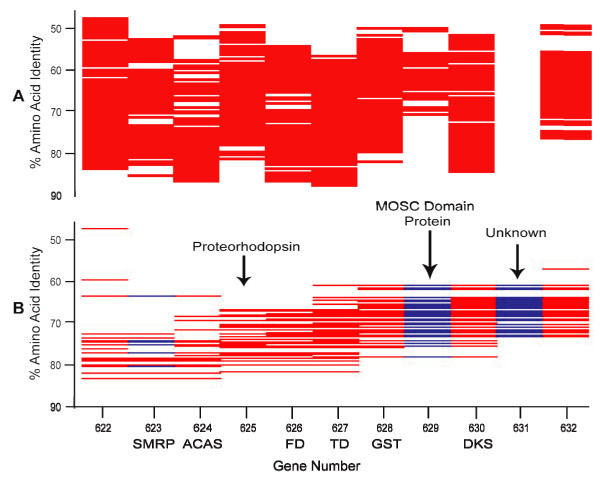
**Detail of Figs 3C and 3D, in the vicinity of the proteorhodopsin gene**. A) homologous fragments that passed the best-hit, and B) syntenic fragments. Regions of blue on the fragments indicate gaps. Only syntenic fragments containing 3 or more genes are shown. (SMRP) small multi-drug resistance protein, (ACAS) acyl-coenzyme A synthetase, (FD) ferrodoxin, (TD) thioredoxin disulfide reductase, (GST) glutathione S-transferase, (DKS) DnaK suppressor protein.

Scrutinized in detail, the syntenic fragment plots reveal many conserved differences between the HTCC strain genomes and the SAR11 metagenome. For example, the syntenic fragment plots illustrate that the Sargasso Sea SAR11 genomes include proteorhodopsin genes (Fig. 5A,B). Proteorhodopsins are light-dependent proton pumps that are hypothesized to provide an alternative energy source for bacterioplankton cells [22,23]. Many of the genes surrounding the Sargasso Sea SAR11 proteorhodopsins are syntenic with the same regions of the HTCC1062 genome, but the syntenic fragment plots indicate that there are two conserved gene deletions downstream of the PR gene in the metagenomic data (Fig. 5B). Reference to the plot of "homologous fragments that passed the best-hit test", above (Fig. 5A), shows that the MOSC binding protein (CDS 629) is found consistently elsewhere in the SAR11 metagenome, but no close homologs to the other "gene indel", CDS 631, were detected.

Nowhere in this analysis did we see clear evidence of the Sargasso Sea SAR11 ecotypes reported previously from this dataset [12], and suggested by Fig. 1. This is not to say that there are no patterns in the plots, particularly the syntenic fragment plots, related to ecotypic variation. Our interpretation is that the dominant two SAR11 ecotypes represented by the metagenomic data are too similar in core genome regions to be discriminated by the approaches we employed, and are best detected by phylogenetic analyses such as that shown in Fig. 1.

### Hypervariable regions

**Figure 6:**
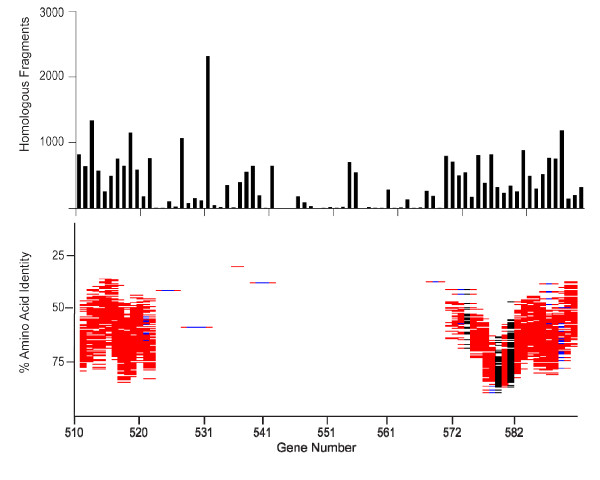
**Enlargement of HVR2**. HTCC1062 syntenic fragment plot showing detail in the region of HVR2.

Although the distributions of homologous fragments and syntenic fragments suggest a relatively conserved SAR11 "core" genome, they also revealed four distinct hypervariable regions, HVR1 – 4 (Fig. 3A,C, Fig. 6, Additional files 7 and 8). Both gene order (synteny) and sequence similarity drop dramatically in the SAR11 hypervariable regions, causing them to stand out prominently as gaps in the plots of homologs and syntenic fragments (Fig. 3A,C, Fig. 6, Additional files 7 and 8). Rusch saw evidence of the same HVRs in "recruitment plots" [13]. "Islands" of genome variability similar to the HVRs we report have been found in many microbial genomes [19,24]. They have been shown to include genes potentially involved in pathogenicity [25] and lipopolysaccharide (LPS) biosynthesis [26]. Most evidence for large microbial pan-genomes comes from variable genomic islands.

The largest of the SAR11 hypervariable regions, HVR2, is a 48 kb region flanked by the sole 5S and 23S rRNA genes in the HTCC1062 genome. HVR2 mainly contains proposed lipopolysaccharide (LPS) biosynthesis genes (Table 5), and appears to be analogous to previously observed regions encoding cell surface properties. Based on current annotations, all but one of the enzymes involved in the biosynthetic pathways for the inner and outer core of lipopolysaccharide (LPS) are present in HVR2, while the enzymes involved in synthesis of the unexposed regions of the LPS are found elsewhere in the genome (Table 5).

**Table 5:** HVR2 genes.

Gene number	Gene product	Functional category	TM helices	Non-syntenic hits
SAR11_0524	unknown	unknown		0
SAR11_0525	nucleotide sugar epimerase	CHO metabolism		1
SAR11_0526	nucleotide sugar epimerase	CHO metabolism		3
SAR11_0527	acetolactate synthase	aa metabolism		5
SAR11_0528	methyl transferase	aa metabolism		22
SAR11_0529	alcohol dehydrogenase	cho metabolism		11
SAR11_0530	phospholipid synthase	om		6
SAR11_0531	short-chain dehyrogenase	CHO metabolism		4
SAR11_0532	oxidoreductase	unknown specificity	1	0
SAR11_0533	methyltransferase	unknown specificity		2
SAR11_0534	aminotransferase	unknown	2	3
SAR11_0535	unknown	unknown	1	0
SAR11_0536	tktc	pentose-phosphate		5
SAR11_0537	tktn	pentose-phosphate		11
SAR11_0538	nucleotide sugar epimerase	CHO metabolism		34
**SAR11_0539**	carbohydrate kinase	CHO metabolism		47
SAR11_0540	glycosyl transferase	CHO metabolism	2	2
SAR11_0541	nucleotide sugar dehydratasez	nt-CHO metabolism		11
SAR11_0542	unknown	unknown	9	0
SAR11_0543	unknown	unknown	3	0
SAR11_0544	unknown	unknown	4	0
**SAR11_0545**	phosphoheptose isomerase	CHO metabolism		10
**SAR11_0546**	sugar phoshatase	om		4
SAR11_0547	amino-acid oxidase	aa metabolism		15
SAR11_0548	unknown	unknown	10	0
SAR11_0549	unknown	unknown	10	0
**SAR11_0550**	glycosyl transferase	om		9
SAR11_0551	probable phage integrase	unknown		3
SAR11_0552	glycosyl tranferase	om		2
SAR11_0553	amino transferase	om		5
SAR11_0554	carbamoyl transferase	antibiotic synthesis		57
SAR11_0555	phospholipid synthase	om		0
**SAR11_0556**	glycosyl tranferase	om		2
SAR11_0557	glycosyl tranferase	om	3	2
SAR11_0558	phospholipid synthase	om		0
SAR11_0559	trehalose phosphate synthase	om		0
SAR11_0560	methyl transferase	unknown		0
SAR11_0561	oxidoreductase	phytobilin synthesis		1
SAR11_0563	glycosyl transferase	cho metabolism		4
SAR11_0564	unknown	unknown		1
SAR11_0565	unknown	unknown		13
**SAR11_0566**	o-antigen polymerase	om	10	40
SAR11_0567	unknown	unknown	11	55
SAR11_0568	oxidoreductase	aa metabolism		1

Bold font indicates genes involved in inner and outer core lipopolysaccharide (LPS) biosynthesis. Genes involved in the biosynthesis of the unexposed (lipid-A portion) of LPS are not present in HVR2. (om) outer membrane; (CHO) carbohydrate; (aa) amino acid

The existence of a hypervariable region similar to HVR2 in the Sargasso Sea SAR11 population was inferred from the metagenomic data. Only two fragments containing SAR11 16S rRNA genes are found among the 349,742 homologous fragments, but SAR11 5S rRNA genes were found on 36 fragments. Nineteen of these fragments carried homologs to HTCC1062 CDS 570 upstream of the 5S rRNA gene, as found in the HTCC1062 genome, suggesting that the Sargasso Sea SAR11 cells, like the Pelagibacter isolates, have split ribosomal RNA operons.

The remaining SAR11 HVR's appear to be related to transport and secretion (HVR1&4) or unknowns (HVR3) (Tables 6, 7, 8, 9, and 10). The transport and secretion functionality associated with HVRs 1 and 4 is consistent with the assertion of Coleman [19] that these islands may play a role in niche adaptation by supporting differential nutrient acquisition capabilities.

**Table 6:** Gene content of HVR2 (523–568), by functional category.

Functional Category	# genes
Cell envelope biogenesis, outer membrane	23
Amino Acid, Carbohydrate, or Nucleotide Transport	7
Defense Mechanisms	2
Export of O-antigen and teichoic acid	2
Signal Transduction	1
Unknown	3
Other	12

**Table 7:** HVR1 genes

Gene number	Gene product	Functional category	TM helices	Non-syntenic hits
SAR11_0042	autotransporter	secretion	1	14
SAR11_0043	unknown	unknown		0
SAR11_0044	autotransporter	secretion	1	10
SAR11_0045	unknown	unknown	1	17
SAR11_0046	autotransporter	secretion	1	25
SAR11_0047	lexa/cap	transcription regulation		7
SAR11_0048	sodium symporter	ion transport	8	218
SAR11_0049	ammonium transporter	ion transport	11	3
SAR11_0050	ammonium transporter	ion transport	11	3
SAR11_0051	metal ion tranporter	ion transport	1	0
SAR11_0052	unknown	unknown		10
SAR11_0053	putative pseudo-pilin pulg	pilin/secretion	1	28
SAR11_0054	pilin	pilin	1	0

**Table 8:** HVR3 genes

Gene number	Gene product	Functional category	TM helices	Non-syntenic hits
SAR11_1162	unknown	unknown		0
SAR11_1163	unknown	unknown		3
SAR11_1164	unknown	unknown	1	24
SAR11_1165	unknown	unknown		0
SAR11_1166	unknown	unknown		0
SAR11_1167	unknown	unknown		2
SAR11_1168	unknown	unknown		0
SAR11_1169	unknown	recb-like		17
SAR11_1170	unknown	unknown	2	0
SAR11_1171	oxidoreductase	c metabolism		27
SAR11_1172	osmc – like	osmotically induced		1
SAR11_1173	betaine-homocysteine methyltransferase	aa metabolism		103

**Table 9:** HVR4 genes

Gene number	Gene product	Functional category	TM helices	Non-syntenic hits
SAR11_1214	possible type iii secretion	Secretion		28
SAR11_1215	sulfotransferase	Unknown		12
SAR11_1216	pilin precursor	Pilin		1
SAR11_1217	bacterial-like globin	Unknown		0
SAR11_1218	phosphatase	sigb regulator		0
SAR11_1219	pilin	Pilin		0
SAR11_1220	unknown	Unknown	1	0
SAR11_1221	sarcosine dehydrogenase	aa metabolism		103

**Table 10:** HVR gene content summary

HVR	Dominant Category	Genes	TM Spanning
1	Transport/Secretion	13	10
2	Outer Membrane/CHO Metabolism	44	12
3	Unknowns	12	2
4	Transport/Secretion	8	1

**Figure 7:**
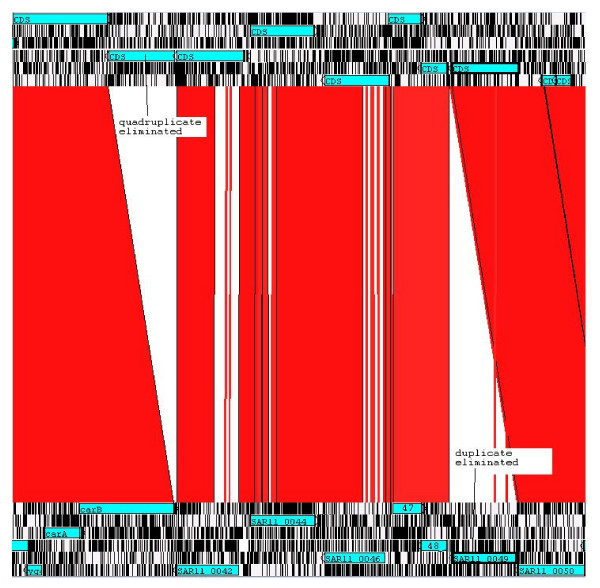
**Deletion of duplicate genes in the HVR1 region of strains HTCC1002 and HTCC1062**. Strain HTCC1002 appears at the top of the display and HTCC1062 at the bottom. One of four homologous Type V autotransporters is deleted in HTCC1062 relative to HTCC1002, and one of two homologous ammonium transporters is deleted in HTCC1002 relative to HTCC1062.

Comparison of the genomes of HTCC1062 and HTCC1002, which were isolated from the same seawater sample [14], provided further support for the conclusion that some HVRs are hotspots for the acquisition of foreign DNA by horizontal gene transfer (HGT). The genome of HTCC1002 is 12,298 nucleotides larger than the genome of HTCC1062. Most of the length difference is due to 31 genes inserted in HVR3 of HTCC1002. The 16S rRNAs of these strains differ by one nucleotide, and in protein coding regions they are 97.4% similar in nucleotide sequence. Although HGT is clearly a source of variation in the HVRs, gene duplications were also observed in the comparison of HTCC1002 and HTCC1062, suggesting that other mechanisms of genome evolution might be contributing the high variability observed in these genome regions (Fig. 7).

### Gene indels in core regions of the HTCC1002 and HTCC1062 genomes: could they be polymorphic sites?

Given the high conservation of core regions of the genome suggested by the plots of syntenic fragments, we were at first surprised to find, in comparisons of the HTCC1062 and HTCC1002 genomes, 62 gene indels (gene insertions in one genome relative to the other) in core regions.

**Figure 8:**
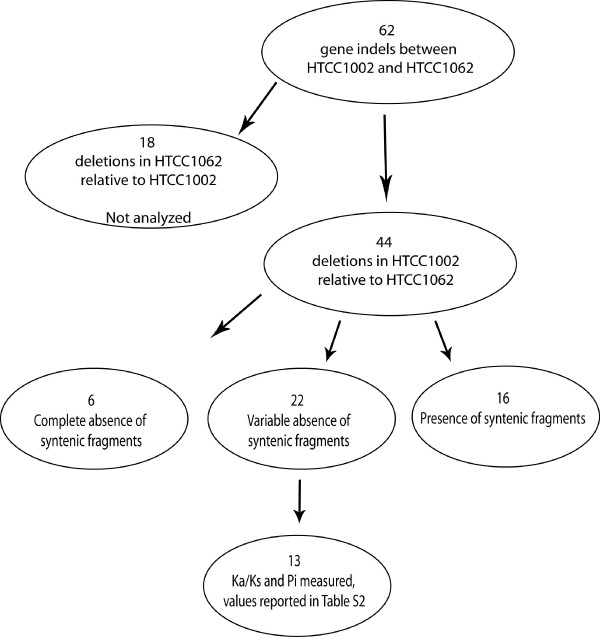
**Summary of analysis of HTCC1002/HTCC1062 "gene indels" in core regions**. The flow diagram shows how 62 HTCC1002/HTCC1062 gene indels and the data from the syntenic fragment plot were combined to choose 13 genes for the tests of selection data shown in Table 4.

**Figure 9:**
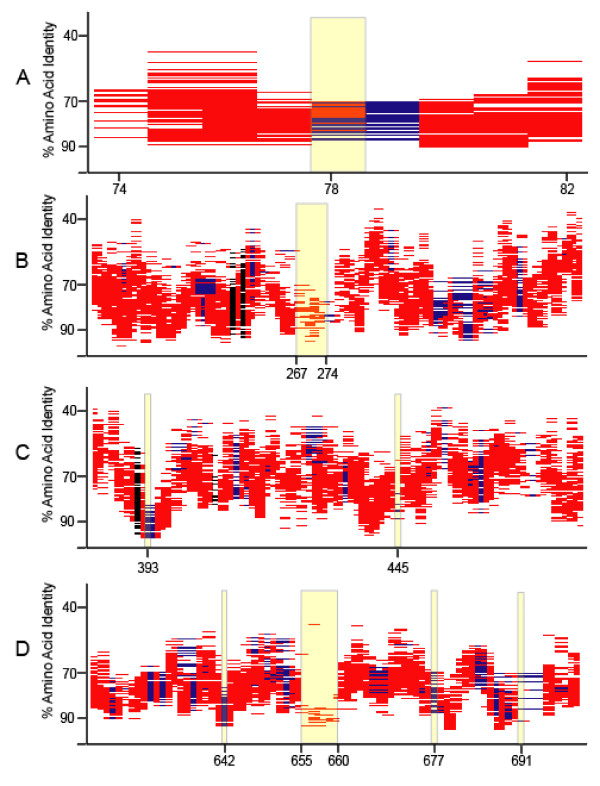
**Syntig coverage of HTCC1002/HTCC1062 deletions**. Shaded areas highlight the syntig coverage above the genes in HTCC1062 that are deleted in closely related strain HTCC1002. Panels A-G show all 44 genes of this type with HTCC1062 gene numbers on the x-axis.

Various data support the model that cells normally harbor a transient pool of neutral genes that are continually culled by a deletion bias in DNA replication [27-29]. The prediction is that random gene insertions will be "neutral" in the sense that they are under no (or low) evolutionary pressure. Syntenic fragment plots provide a test of this model, since the prediction is that there will be no coverage in the column above genes that are randomly inserted into the query genome. Of 62 indels between the two genomes, 44 are deletions in HTCC1002 relative to HTCC1062, and are thus testable with the HTCC1062 sytenic fragment plot (Fig. 8). In 28 of 44 cases, syntig coverage of these genes was either entirely or partially absent (6 and 22 cases respectively), and in cases where sytenous fragments were present, the coverage is either light, highly divergent, or both (Fig. 9). These observations indicate that most of the gene indels found in core regions of the HTCC1062 and HTCC1002 genomes have a very atypical appearance in the syntenic fragment plots, in comparison to most genes found in the core regions of the Pelagibacter genomes. Some appear to be entirely absent, suggesting that they might be random gene insertions, while others are variably present in the metagenomic data. Ka/Ks values for the set of HTCC1002/1062 gene indels that were variably present indicate that these genes are subject to purifying selection (Table 4).

Previous studies of *E. coli *in culture reported a balanced polymorphism that arose spontaneously and was maintained by selection [30]. The resulting haplotypes diverged phylogenetically and met Cohan's definition for ecotypes [31]. Thus, there is precedent that anticipates balanced polymorphisms in natural microbial populations. We speculate that the gene indels we observed could be balanced polymorphisms, and that this phenomenon might explain part of the natural variation in gene content found in microbial populations.

### Gene complement

We were surprised to find no indication of conserved differences in gene complement that would suggest significant physiological differences between the coastal strains and Sargasso Sea SAR11 populations. This is not to say that there is not physiological variation within and between the Sargasso Sea and coastal Oregon SAR11 populations. Rather, we observed no genes thought to control significant phenotypic attributes in HTCC1002 and HTCC1062 that were not also well-represented among the syntenic fragments, and no conserved occurrences of functionally important genes in the SAR11 metagenomic data that did not have orthologs in the HTCC1062 genome. We should have seen such genes if they were highly conserved, because our analysis included an average of 118 fragments that covered each end of each gene and provided evidence for the identity of the adjacent gene. Only 19 genes from the HTCC1062 genome are not represented in the SAR11 metagenome (syntenic fragments or homologous fragments that passed the best BLAST hit test). Of these, nine are from the hypervariable regions (Table 11), and six returned no hits to NCBI databases with expect scores less than 1 × 10^-10^. Two members of this group (CDSs 542 and 555) are suspected to be involved in outer membrane biosynthesis and CDS 1217 is a bacterial-like globin (Table 11). With only one exception, we found closer homologs to these genes in the Sargasso Sea dataset than in NCBI databases.

**Table 11:** Evidence for genes specific to the coastal variants of SAR11.

Gene number	Gene annotation	SSD	NCBI	HVR
SAR11_0043	unknown protein*	4	None	1
SAR11_0163	unknown protein*	1 × e^-10^	1.6	
SAR11_0414	unknown protein*	1 × e^-4^	None	
SAR11_0471	unknown protein*	3 × e^-9^	2.1	
SAR11_0542	unknown protein*	3 × e^-10^	5 × e^-11^	2
SAR11_0544	unknown protein, possible viral origin	3 × e^-6^	3 × e^-4^	2
SAR11_0548	unknown protein	8 × e^-9^	6 × e^-5^	2
SAR11_0555	unknown protein	3 × e^-8^	6 × e^-4^	2
SAR11_0631	unknown protein*	1.1	None	
SAR11_0788	unknown protein*	9 × e^-5^	None	
SAR11_0875	unknown protein	1 × e^-10^	None	
SAR11_0930	unknown protein*	0.47	1.2	
SAR11_0989	unknown protein	0.61	None	
SAR11_1165	unknown protein, possible exonuclease	4 × e^-7^	1.3	3
SAR11_1170	unknown protein	1.8	2.8	3
SAR11_1182	unknown protein	3 × e^-10^	2.2	
SAR11_1217	bacterial globin-like	4 × e^-10^	2 × e^-8^	4
SAR11_1220	unknown protein	2 × e^-4^	6.2	4
SAR11_1249	unknown protein	2 × e^-6^	3.7	

This list includes all genes for which no homologs were found in the Sargasso Sea Dataset (SSD) with a TBLASTN expect score of less than or equal to 1 × e^-10^. There were no examples of genes with known, significant physiological functions in this category. The best TBLASTN expect score against the Sargasso Sea Dataset (SSD) and the NCBI non-redundant nucleotide database (NCBInr), and the hypervariable region (HVR) in which the gene was found, are listed. Astericks indicate short CDS that may be gene calling error.

## Conclusion

The reconstruction of microbial genomes from metagenomic data is a challenge for microbial ecologists, particularly when the genomes originate from large plankton populations that exhibit inherently high natural variability. Venter attributed the failure to assemble SAR11 genomes for the Sargasso Sea metagenomic data, despite apparently high coverage, to high species diversity. But, an alternative explanation is that some genomes are inherently diverse in neutral characteristics because of large effective population sizes. In such cases, it seems likely that a linear genome, the objective of assembly, is an overly simplistic model for representing natural genome variation. Therefore, we sought a strategy that would reveal conserved and variable elements of genome structure. This strategy was predicated on the use of a related query genome, and designed to be useful for predicting genome properties for studies of ecology and evolution.

The amino acid sequence divergence between the query genome we used and the Sargasso Sea SAR11 populations exceeds the divergence between some microbial genera, suggesting that genomic properties have had ample time to diverge in response to selection. We were surprised to find that there was heavy coverage of most of the genome by syntenic fragments, suggesting a relatively conserved gene order in core regions of the genome. Previous reports have shown that the conservation of gene order between prokaryotic genomes dissipates faster than protein sequence identity or gene complement [32,33]. Synteny is regarded as a rapidly evolving property of genomes, second only to DNA with regulatory functions [34]. Huynen and coworkers compared orthologs from an evolutionarily diverse set of 9 genomes to show that gene order becomes nearly random before protein identity decays below 50% [34].

The seemingly incongruous observation of the preservation of synteny in the presence of 30% divergence in average amino acid sequence can be explained by noting that Huynen and coworkers studied species that are highly divergent in functional properties, whereas the SAR11 population appears relatively uniform, despite the accumulation of considerable neutral sequence variation. This explanation implies that selection is acting to preserve gene order in SAR11 populations. We speculate that this may be another example of streamlining selection, in this case acting to preserve gene order [9].

Rusch et al. [13] reported that synteny was conserved in all abundant marine prokaryotes, but their method of observing synteny was qualitative and therefore they did not offer a quantitative estimate that could be used for comparative purposes. We provide a quantitative estimate of synteny and also show that genome rearrangements are not random, but are concentrated at particular sites, which are often operon boundaries. This observation has implications for the "extreme assembly" employed by Rusch, which has an unproven ability to detect and report the frequency of genome rearrangements.

The high coverage provided by this dataset enabled us to examine core regions of the genome for conserved gene rearrangements, including insertions. Although many conserved gene rearrangements were observed, none involved genes acquired by HGT. The approaches we describe are robust for some conclusions; for example, the identification of genomic regions that are missing or highly diverged from the query sequence, and regions, such as the proteorhodopsin gene locus, where genes and gene order vary in conserved patterns. There are also caveats. For example, it is likely that only the termini of large cassettes of inserted genes in the target genomes can be observed, and then, only where they abut regions of conserved gene order. Thus, the absence of any observations suggesting conserved insertions of novel genes in the SAR11 metagenomic data suggests that the Sargasso Sea SAR11 variants are very similar to their coastal counterparts, in core regions of the genome, but does not rule out the involvement of HVRs in evolution by gene acquisition.

The concept of the "core" and "pan" genomes is gathering support as genome sequencing reveals more examples of conserved core genome regions and hypervariable regions, or islands of genome variability, as they are sometimes known [3,19]. While the mechanisms that generate variability in these regions remain poorly understood, it is increasingly apparent that they often encode niche-specific proteins that are important to competitive success. Our observations are consistent with the interpretation that natural selection has concentrated genes that encode cell surface properties into HVR2, and that this region is subject to unusually rapid rates of sequence divergence and re-arrangements of gene order. It has been postulated that similar variability in the *C. jejuni *LPS cassette is an adaptive response to selection pressure to evade host acquired immune responses [35]. Viral predation on microbial cells is intense in the ocean water column and is likely to provide a keen source of selective pressure that favors microbial populations with diverse, rapidly evolving surface properties. An analogous variable genome region containing genes for cell surface components (LPS cassettes) has been observed in *Prochlorococcus sp*. [26,36], and is evident in our syntenic fragment plot for this organism (arrow in Additional file 4B). We propose that the structural RNA genes flanking the LPS cassettes provide zones of conserved DNA sequence that promote horizontal exchange of the cassettes by homologous recombination. Multi-locus sequence typing has shown that rates of intraspecific recombination are high within the coastal SAR11 population [37]. Alternatively, this variable genome region could be explained by horizontal gene transfer from another species, a hypothesis that is consistent with the observation that the AT content of HVR2 is anomalously high (79%).

To explain the apparent conservation of core genome regions, and reconcile it with the apparent variability of SAR11 genomes in nature, we hypothesize that the large population sizes of SAR11, and the age of these clades that is inferred from phylogenies, have allowed them to accumulate very extensive variation in genome properties that are selectively neutral [2]. However, it is clear that our study offers only a glimpse of the evolution of the SAR11 clade. In particular, the study of genome organization, as inferred from plots of syntenic fragments and other methods, revealed less about the evolution of SAR11 ecotypes than did phylogenetic analyses. In part, we attribute this to a relatively high level of conserved organization in SAR11 genomes, and the role of hypervariable regions, which by definition are not amenable to studies based on the identification of conserved genome features.

An intriguing finding is the correspondence between "gene indels" in core regions of the two Pelagibacter genomes, and patterns in the synteny plots from another ocean. This finding needs further investigation. In part, it is consistent with the established idea that genes are randomly inserted into genomes at a low frequency, and lost if not preserved by positive selection [27-29]. But, our findings suggest that some gene indels might have another explanation. They could be polymorphisms that are maintained in populations by processes similar to those that maintain allelic polymorphisms [30]. The most appealing approach to resolving this interesting question would be the sequencing of many genomes from closely related isolates.

Our findings indicate that SAR11 genomes from different oceanic provinces share many conserved features despite dynamic processes of genome change that are at work in nature. The Sargasso Sea SAR11 populations are conserved in local gene order, and gene complement, with respect to populations that live in richer, colder coastal water, but diverge dramatically in amino acid sequence similarity. A broad implication is that large microbial populations such as bacterioplankton accumulate high diversity in some genome properties, while remaining constrained in others [2]. Protein evolution provides an analogy. Protein families can encompass wide variation in amino acid sequences while retaining the key elements of three-dimensional structure that confer function [38]. Similarly, in old clades that comprise large populations, microbial genomes may wander over sequence space, giving an illusion of variability, while remaining highly constrained in features that govern cellular structure and function. An important challenge going forward will be in establishing quantitative measures that reveal these properties of genome conservation from metagenomic data.

## Materials and methods

### Phylogenetic analysis

Amino acid sequences were aligned with ClustalW [39] (gap open penalty: 100; gap extension penalty: 0.2). Maximum likelihood trees were constructed with RAxML using the PROTMIXBLOSUM62 amino acid model. Bootstrap values are based on 100 iterations [40].

### Homolog search

Fragments carrying genes with high similarity to HTCC1062 genes are identified at the protein level with TBLASTN [41], using the amino acid sequence as input, a 1 × 10^-10 ^expect score cutoff, and complexity filtering off. The results are limited to the first 3,000 hits. Command line: blastall -i sar11_proteins.fa -d venter_nt -p tblastn -e '1e^-10^' -F F -v 3000 -b 3000, where sar11_proteins.fa is a fasta file of HTCC1062 proteins, venter_nt is the Sargasso Sea fragment data in BLAST format. Default values were used for all unnamed parameters, blastall version was 2.2.12. For convenience, the set of fragments identified in this fashion are hereafter referred to as "homologous fragments".

### Syntenic fragment detection

A subset of the homologous fragments that shared synteny with the HTCC1062 genome was identified by finding fragments that were common to the lists from adjacent HTCC1062 genes, and verifying that the genes are arranged in tandem on the fragment. Each gene on these syntenic fragments was subjected to the reciprocal best-hit test (Fig. 2). The fragment nucleotide sequence of the high-scoring sequence pair (HSP) from the TBLASTN search for homologs was searched against the NCBI non-redundant proteins database using BLASTX. The accession number of the best hit was compared to the accession number of the predicted HTCC1062 gene to confirm the identity of the reciprocal best hit. The term "syntenic fragment" thus designates fragments containing at least two best-hitting HTCC1062 genes in the proper order, but does not itself indicate anything about the rest of the fragment, for instance the presence of genes without homologs in HTCC1062.

### Syntenic fragment plots with different query genomes

To assess the selectivity of syntenic fragments we studied a set of organisms of varying relevance to ocean surface ecology. Of these, *Procholorococcus marinus *MED4 provides an example from a clade that is relatively abundant in the Sargasso Sea but forms a shallow cluster by 16S rRNA gene sequence analysis [42]. *Escherichia coli *was chosen as an organism that is unlikely to appear often in the Sargasso Sea. Five additional cultured marine strains were also used as query genomes. They are listed in Table 2. We found that the syntenic fragment plots are characteristic for each group, and the numbers of syntenic fragments in the plots correlates with the abundance of each organism's 16S rRNA genes in the metagenomic data (Additional file 4). For each selected organism, the relative abundance of genomic DNA within the Sargasso Sea metagenome is estimated by the number of 16S rRNA fragments satisfying identity thresholds of 97%, 93%, and 90%. These values are shown as insets in Additional file 4. The number of syntenic fragments recovered for each organism correlates with the number of similar 16S rRNA genes. The most abundant organism, SAR11, produced by far the most syntenic fragments (71,696) while *P. marinus *returned an intermediate number (8,398). Other organisms (e.g. *C. atlanticus *in Table 2 and Additional file 4) that are virtually undetectable by 16S rRNA analysis returned a number of syntenic fragments (1258) similar to our negative control *E. coli *(406). Additional file 3 shows the distribution of syntenic fragments recovered for *E. coli*.

If the syntenic fragment detection process described here accurately recovers environmental fragments arising from a given template organism, then the list of syntenic fragments should be unique for each organism. Thus, to estimate the selectivity of our method we compared the syntenic fragment lists of all organisms tested. The percentage of syntenic fragments unique to each organism (Table 2) is 98.8 % for HTCC1062 and 99.0 % for *P. marinus*. Less-abundant organisms have as few as 30% unique syntenic fragments, but not in all cases. In numbers, the low abundance organisms are not distinguishable from each other or *E. coli*, though other marine microbes show a narrower, more distinct syntenic fragment pattern than *E. coli*, similar to Additional file 4C (data not shown).

### Genome rearrangements and operon prediction

To find fragments containing HTCC1062 genes in altered gene orders, the genes on the homologous fragments that did not show synteny to HTCC1062 were subjected to the best BLAST analysis described above for the syntenic fragments. The number of occurrences of a given best-hitting HTCC1062 gene adjacent to a non-syntenic best-hitting HTCC1062 gene was determined, scoring the number of times any unique pair of genes occurred together.

To determine if genome rearrangements are concentrated at operon boundaries, a statistical analysis was performed comparing the number of gene pairs found that violate operon boundaries (disallowed pairs; n = 1301), to the number found that preserve operon boundaries (allowed pairs; n = 2131). A maximum likelihood estimator was used to calculate the probability that this observation could be due to chance, assuming the rearrangements at gene boundaries follow a normal distribution. The list of predicted operons for P. ubique HTCC1062 was obtained from via the ENTREZ Genome Project [21].

### Hypervariable regions

Site-specific recombination mediated by integrases has been shown to cause rapid change in some islands of genomic variability [24]. However, SAR11, as with most examples thus analyzed [43], failed to display clear signatures of the integron model – attC sites were not found associated with any of the HVRs. HVR2 includes an integrase gene, and HVR4 is flanked by tRNA genes, which have been shown to serve as site-specific recombination sites for temperate phages and transmissible plasmids [44].

Comparison of the genomes of strains HTCC1062 and HTCC1002 provides evidence that the 23S and 5S rRNA genes flanking HVR2 are sites of homologous recombination that allow novel variations of the LPS region to spread rapidly within populations. The HVR2 regions in these two genomes are 99.96% similar in nucleotide sequence, compared to 97.4% similarity for the genomes overall. In addition to few point mutations, the two HVR2 sequences differ by a deletion of 13 nucleotides that removes one from a set of four tandem repeats within a hypothetical gene.

In contrast, the HVR1 regions of HTCC1062 and HTCC1002 reveal the loss and gain of divergent, tandem duplicated genes. One gene is deleted from a set of four tandem, divergent gene duplications of Type V secretion proteins in strain HTCC1062 (Fig. 7). In strain HTCC1002, a single ammonium transporter gene is deleted from two, tandem duplicated genes. A high proportion of hypothetical genes, such as we found in the SAR11 hypervariable regions, is a general feature of genome islands. While phages are suspected to be the reservoir for this novel gene pool [43] direct evidence for this hypothesis remains elusive. Daubin and Ochman reported that *E. coli *hypothetical genes are short, AT rich, and most likely originate from phage [45]. The evidence for rapid gene evolution by duplication, divergence, domain rearrangements and deletion observed in HVR1 could also be explained by the alternative hypothesis that genetic processes intrinsic to the cell cause at least some of the rapid change in the SAR11 HVRs.

### Tests for selection

Sequences were analyzed for synonymous and non-synonymous substitution rates using the software program SWAAP 1.0.2 [46], set to the Li method (1993) with a window size of 90 and step size of 18. The values reported in Table 2 were created from alignments that include those portions of the HTCC1062 gene and syntenic fragment sequence defined by the HSP start and end positions taken from the TBLASTN results (see homolog detection step). Translated sequence was used to guide the alignments when necessary. Nucleotide divergence values were calculated with the software program DnaSP 4.0 using the 'synonymous non-synonymous substitution' option under the analysis menu with default parameters [47].

### Searching for genes conserved in Sargasso Sea SAR11 populations and not found in the genomes of the coastal isolates

Non-homologous genes found alongside genes that had best hits to HTCC1062 genes were regarded as candidates for genes specific to the Sargasso Sea SAR11 populations. The amino acid sequence of each CDS (the determination of open reading frames on the environmental fragments was taken from the conserved domain feature tags of the NCBI GenBank record) on every homologous fragment with sufficient length to include non-HTCC1062 homologs was used as a BLAST query sequence against the NCBI non-redundant proteins database (BLASTP, expect score cutoff 1 × 10^-6^) and the NCBI Conserved Domain Database (CDD) [48]. We examined the data for the frequency of any specific non-HTCC1062 gene occurring next to a given HTCC1062 gene, using the gene descriptions from the NCBI database as well as the protein family identifier from the CDD as search strings for the identification of common genes.

### Calculating a synteny index

We define a synteny index to be the fraction of best-hitting HTCC1062 homologs found adjacent to a best-hitting and syntenic HTCC1062 homolog. The amino acid sequence of each CDS on all homologous fragments carrying at least two genes (238,663 of 349,742 total, Table 1) was queried against the NCBI non-redundant proteins database. For every best-hitting gene on the fragment, if at least one best-hitting neighbor was present, it was counted as a syntenic observance. To calculate the synteny index the total of all syntenic observances is divided by the total observances of best hitting genes, syntenic and non-syntenic.

### Genes conserved in the coastal isolates but not found in the Sargasso Sea SAR11 populations

HTCC1062 genes that were not represented among the set of homologous fragments identified in the Sargasso Sea dataset by TBLASTN were considered as candidates for genes specific to the coastal ecotypes (Pelagibacter). The translation products of these CDS were used as queries in a TBLASTN search of the Sargasso Sea dataset and the NCBInr database, using an expect score cutoff of 10. The highest scores found for these 19 genes are listed in Table 11. Three CDS (414, 788 and 875) failed to hit the NCBInr database with an expect score of less than 1 × e^-4 ^and are classified as hypothetical proteins in the HTCC1062 genome.

## Competing interests

The author(s) declare that they have no competing interests.

## Authors' contributions

LJW carried out the programming and drafted the manuscript. HJT contributed the comparisons of the HTCC1002 and HTCC1062 genomes. SAG operates the annotation pipeline. DPS carried out the analysis of genome re-arrangements. SJG conceived of the study, and participated in its design and coordination and helped to draft the manuscript. All authors read and approved the final manuscript.

## Reviewer's comments

### Reviewer's report 1

#### Eugene Koonin, National Center for Biotechnology Information, Bethesda, MD

This is quite an exciting study that makes an extensive use of metagenomic data to assess the natural variability of Pelagibacter (SAR11), apparently, the most abundant marine bacterium. Pelagibacter seems to break the trend that, previously, seemed consistent in all compared prokaryotic genomes, namely, that gene order evolves faster than sequence (at least, coding sequence). It is reasonably proposed that the high effective population size of Pelagibacter leads to rapid accumulation of neutral substitutions. It is further proposed that purifying selection acts to maintain the gene order in Pelagibacter and that this is somehow related to genome streamlining. The connection seems pretty mysterious but, at face value, it is hard to deny that, at the time scale when many substitutions accumulate, genome rearrangements do not. An explicit population-genetic analysis of this discrepancy would be interesting but one needs a good model for genome rearrangements, of course.

There seems to be striking analogy between the hypervariable regions in Pelagibacter discovered here and pathegenicity islands in bacterial pathogens, e.g., E. coli. I think it is worth more emphasis.

It will be very revealing to see whether these findings smoothly extend to other marine bacteria such as Prochlorocccus, and whether the population-genetic explanation holds compared to the available abundance data. I actually suspect that the data is there for such a comparison to be done right now but, certainly, that can be done in a new paper, the present one stands on its own.

### Reviewer's report 2

#### Igor B. Jouline (Zhulin), Associate Professor and Senior R&D Staff Member, Joint Institute for Computational Sciences, The University of Tennessee – Oak Ridge National Laboratory

This study aims at evaluating metagenomics data on the most abundant marine proteobacteria, SAR11, by comparing it with available whole genome data of the SAR11 strains. The overarching goal is to understand the nature of species and ecotypes in SAR11 populations. A more specific aim was to assess trends in the SAR11 genome evolution by determining the core genome and variable regions.

One of the main results of this study is a quantitative estimate of synteny and a demonstration that genome rearrangements in SAR11 are not random, but are concentrated at particular sites, which are often operon boundaries.

On a broader scale, this work presents an approach, which is general enough to be applied to other metagenomic data sets in order to study evolutionary trends in natural microbial populations.

I don't have any major concerns regarding this paper. It reads well and I found it quite interesting for a non-specialist in microbial ecology and metagenomics. I would definitely recommend it to anyone who is seriously thinking about deriving information from the metagenomics data sets.

### Reviewer's report 3

#### Peer Bork, senior scientist (bioinformatics)/group leader at the EMBL (Heidelberg); joint coordinator of the EMBL Structural and Computational Biology program; visiting group leader at the MDC (Berlin-Buch)

I think this is a great case study that wisely uses metagenomics data to understand more about the natural variation and evolution of Pelagibacter/SAR11, probably the most abundant marine bacterium.

A number of trends could be observed, some are similar to studies in other phyla, some are different; so the paper also adds in general terms to our understanding of bacterial populations and the impact of habitats on their evolution.

Interestingly, it is proposed that gene order seems to surprisingly evolve slower than sequence and which is explained partially by the large population size. I assume that for the more detailed Quantification of this observation more data will be needed, but the approach demonstrates the power of integrating metagenomics data in genome analysis.

The quantification of the non-random accumulation of mutations along the genome is another interesting aspect worth noting as is a surprizngingly high number of core genes (or relative little indels) given the many individuals from which sequence fragments were available.

Taken together, the paper implies substantial amount of work in an important research area, it is based on a new integration approach, and it addresses an number of important points that are all worth publishing.

## Supplementary Material

Additional file 1 Distribution of expect scores for Pelagibacter syntenic fragments. The values in the plot are taken from the TBLASTN search in which predicted proteins from the HTCC1062 genome were the query genes and the Sargasso Sea metagenomic database was searched (Fig. 2A). The distribution indicates a sharp decline in expect scores approaching the cutoff of 1 × 10^-10^. The data are provided as support for this choice of expect score cutoff. Click here for file

Additional file 2 GC content of Pelagibacter syntenic fragments. Histogram of the GC content of metagenomic fragments from the syntenic fragments bin, compared to the mean for the HTCC1062 genome. Click here for file

Additional file 3 Sargasso Sea syntenic fragment plot for *Escherichia coli*. A syntenic fragment plot using *Escherichia coli *as the query genome. The data provide a rough measure of the false-positive rate in the syntenic fragment plots. Click here for file

Additional file 4 Syntenic fragment plots of three representative organisms. The bar chart in the upper right corner indicates the number of fragments containing the query organism's 16S rRNA, at the indicated degree of similarity. The horizontal line indicates the average syntenic fragment score. Click here for file

Additional file 5 Syntenic fragments carrying at least three genes. A large portion of the syntenic fragments are not of sufficient length to carry more than three genes. These data show that the general trends of genome coverage and range of amino-acid level identity shown in Fig. 3D hold when the shorter fragments are excluded. Click here for file

Additional file 6 Analysis of assemblies versus unassembled reads. Pelagibacter syntenic fragment analysis performed on unassembled reads (A), and on sequence data containing assemblies as well as unassembled reads (B). The plots are essentially similar. Click here for file

Additional file 7 Enlargement of HVR3 and HVR4. HTCC1062 syntenic fragment plot showing detail in the region of HVR3 and HVR4. Click here for file

Additional file 8 Enlargement of HVR1. HTCC1062 syntenic fragment plot showing detail in the region of HVR1. Click here for file
