# Supplementary material for: Natural variation in SAR11 marine bacterioplankton genomes inferred from metagenomic data
Source: Biol Direct. 2007 Nov 7;2:27. doi: 10.1186/1745-6150-2-27 (PMC2217521; doi:10.1186/1745-6150-2-27)
Supplement: Additional file 1 — Distribution of expect scores for Pelagibacter syntenic fragments. The values in the plot are taken from the TBLASTN search in which predicted proteins from the HTCC1062 genome were the query genes and the Sargasso Sea metagenomic database was searched (Fig. 2A). The distribution indicates a sharp decline in expect scores approaching the cutoff of 1 × 10^-10^. The data are provided as support for this choice of expect score cutoff. [file 1745-6150-2-27-S1.doc]

**Distribution of expect scores for Pelagibacter syntenic fragments.**
