# Supplementary material for: Natural variation in SAR11 marine bacterioplankton genomes inferred from metagenomic data
Source: Biol Direct. 2007 Nov 7;2:27. doi: 10.1186/1745-6150-2-27 (PMC2217521; doi:10.1186/1745-6150-2-27)
Supplement: Additional file 2 — GC content of Pelagibacter syntenic fragments. Histogram of the GC content of metagenomic fragments from the syntenic fragments bin, compared to the mean for the HTCC1062 genome. [file 1745-6150-2-27-S2.doc]

**GC content of Pelagibacter syntenic fragments.**

15.6

20.1

24.1

28.1

32.1

36.1

40.6

0

100

200

300

400

500

600

700

% GC

29.7% GC

Syntig mean

29.1% GC

*P.ubique 29.7% GC*

Number of Syntenic Fragments
