# Supplementary figures and images for: Natural variation in SAR11 marine bacterioplankton genomes inferred from metagenomic data
Source: Biol Direct. 2007 Nov 7;2:27. doi: 10.1186/1745-6150-2-27 (PMC2217521; doi:10.1186/1745-6150-2-27)

**Sargasso Sea syntenic fragment plot for *Escherichia coli.***

**
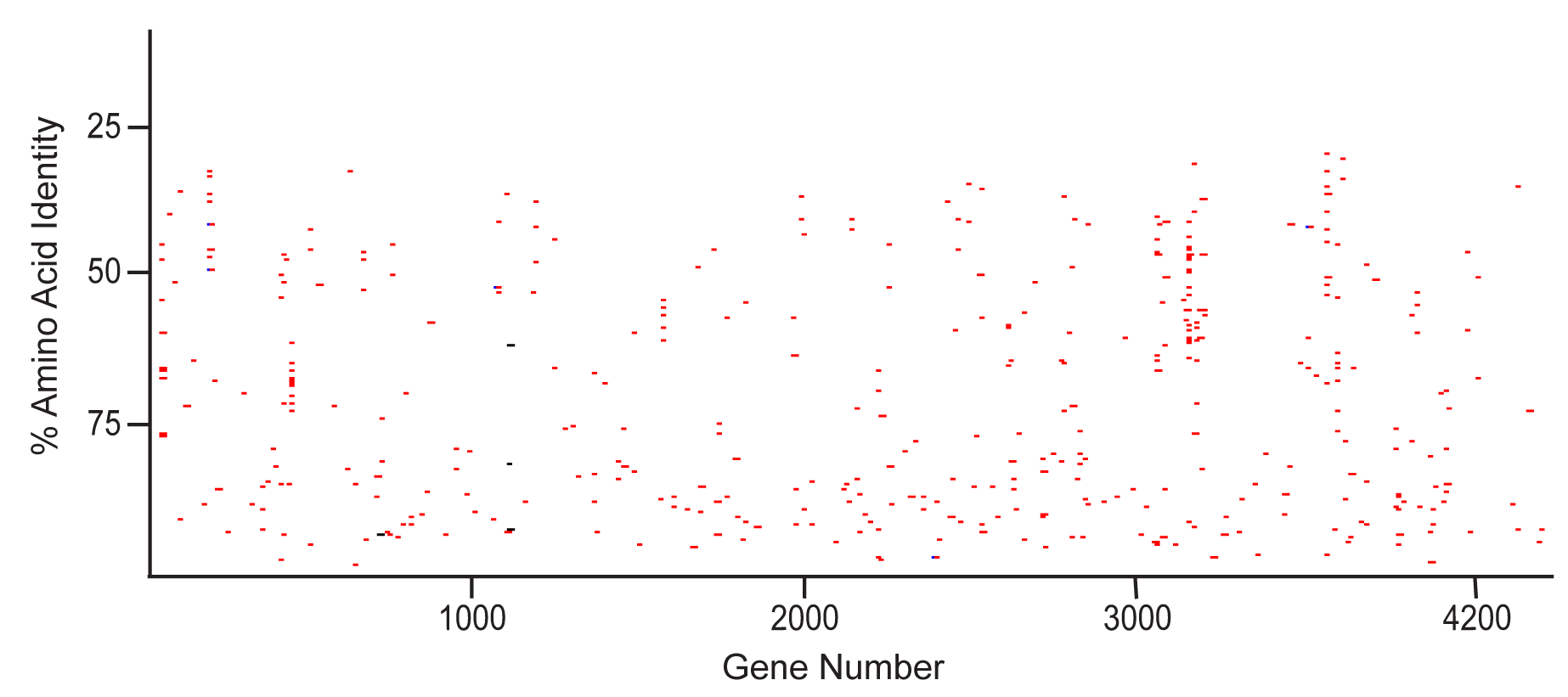
**

Supplement: Additional file 3 — Sargasso Sea syntenic fragment plot for Escherichia coli. A syntenic fragment plot using Escherichia coli as the query genome. The data provide a rough measure of the false-positive rate in the syntenic fragment plots. [file 1745-6150-2-27-S3.doc]

**Syntenic fragment plots of three representative organisms.**

**
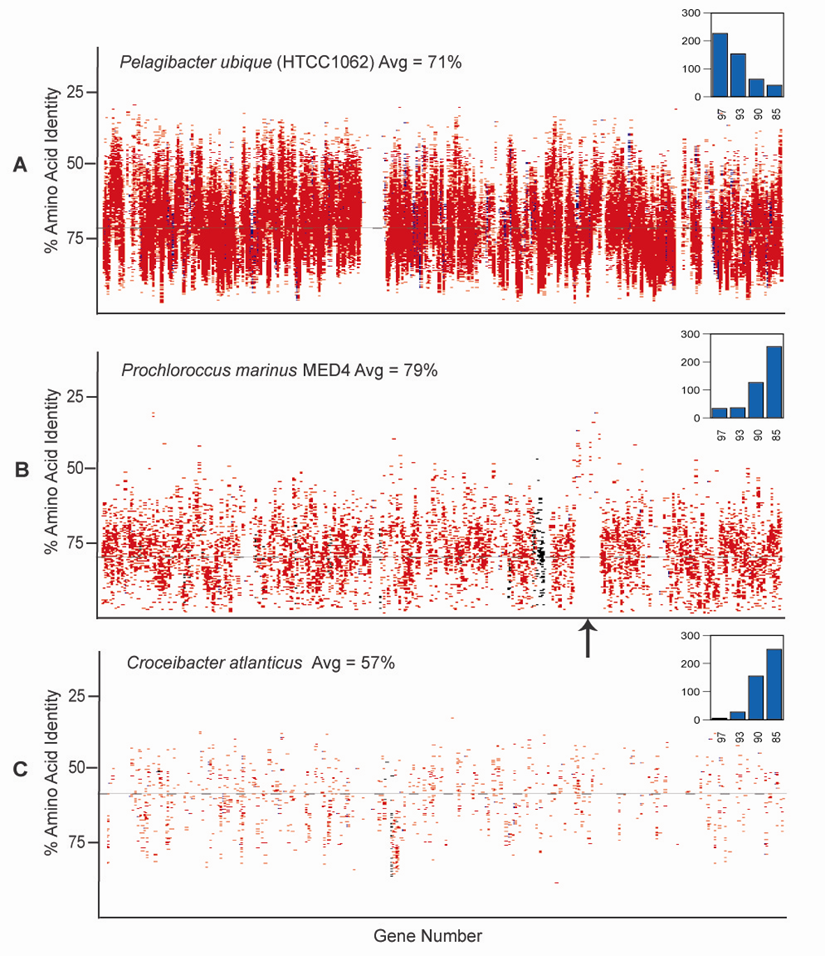
**

Supplement: Additional file 4 — Syntenic fragment plots of three representative organisms. The bar chart in the upper right corner indicates the number of fragments containing the query organism's 16S rRNA, at the indicated degree of similarity. The horizontal line indicates the average syntenic fragment score. [file 1745-6150-2-27-S4.doc]

Pelagibacter syntenic fragment plot showing fragments carrying at least 3 genes.

**
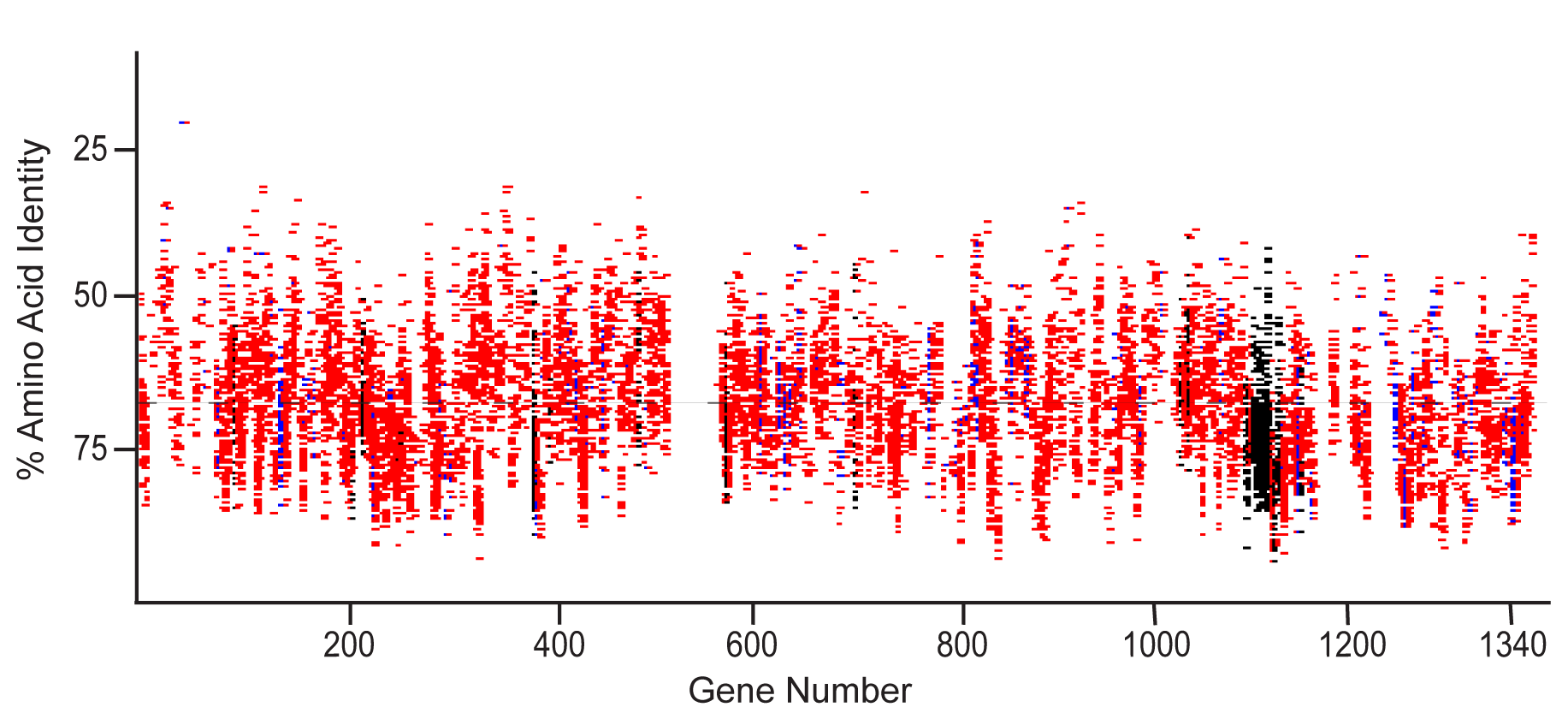
**

Supplement: Additional file 5 — Syntenic fragments carrying at least three genes. A large portion of the syntenic fragments are not of sufficient length to carry more than three genes. These data show that the general trends of genome coverage and range of amino-acid level identity shown in Fig. 3D hold when the shorter fragments are excluded. [file 1745-6150-2-27-S5.doc]

**Analysis of assemblies versus unassembled reads.**

**
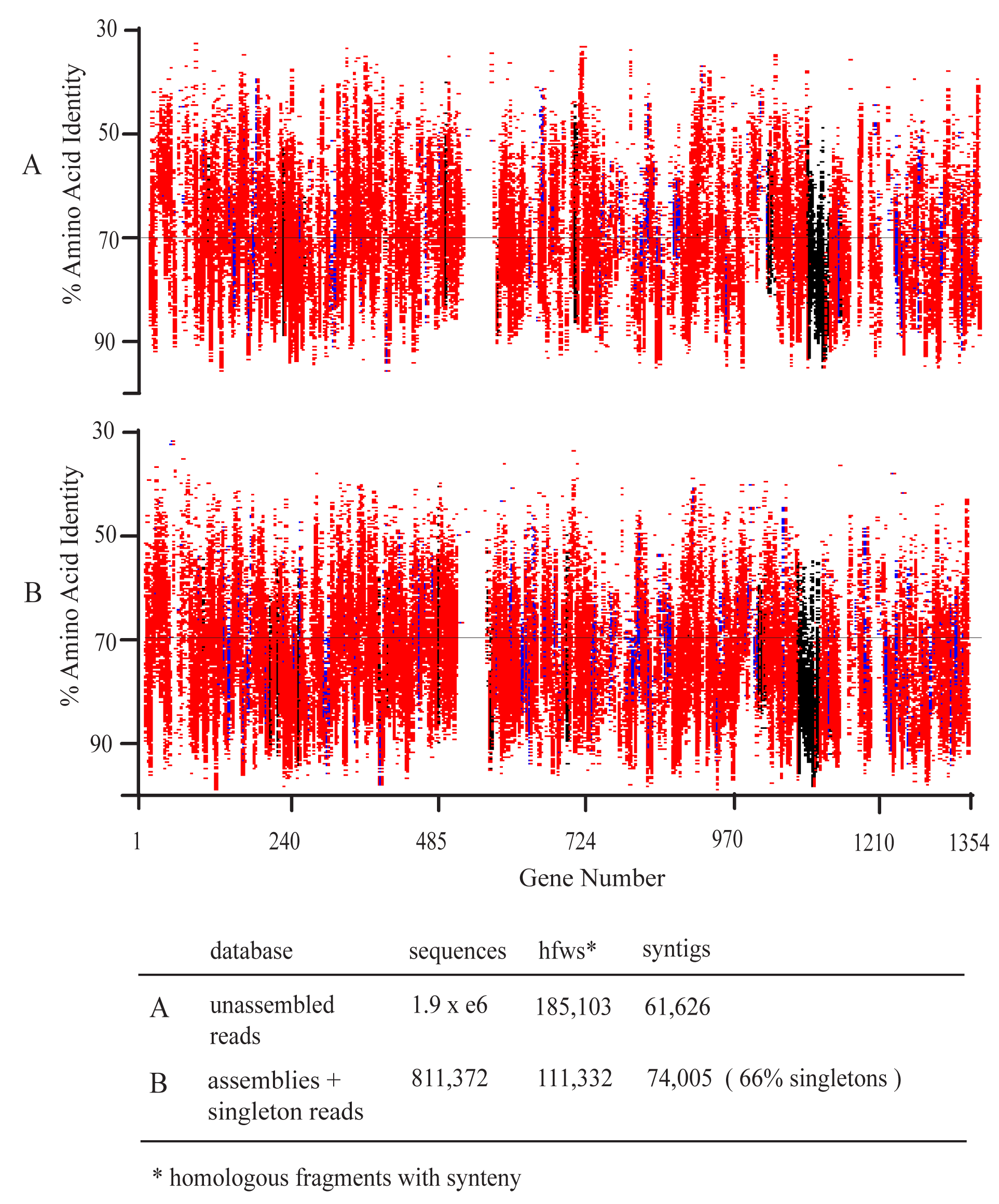
**

Supplement: Additional file 6 — Analysis of assemblies versus unassembled reads. Pelagibacter syntenic fragment analysis performed on unassembled reads (A), and on sequence data containing assemblies as well as unassembled reads (B). The plots are essentially similar. [file 1745-6150-2-27-S6.doc]

**Enlargement of HVR3 and HVR4.**


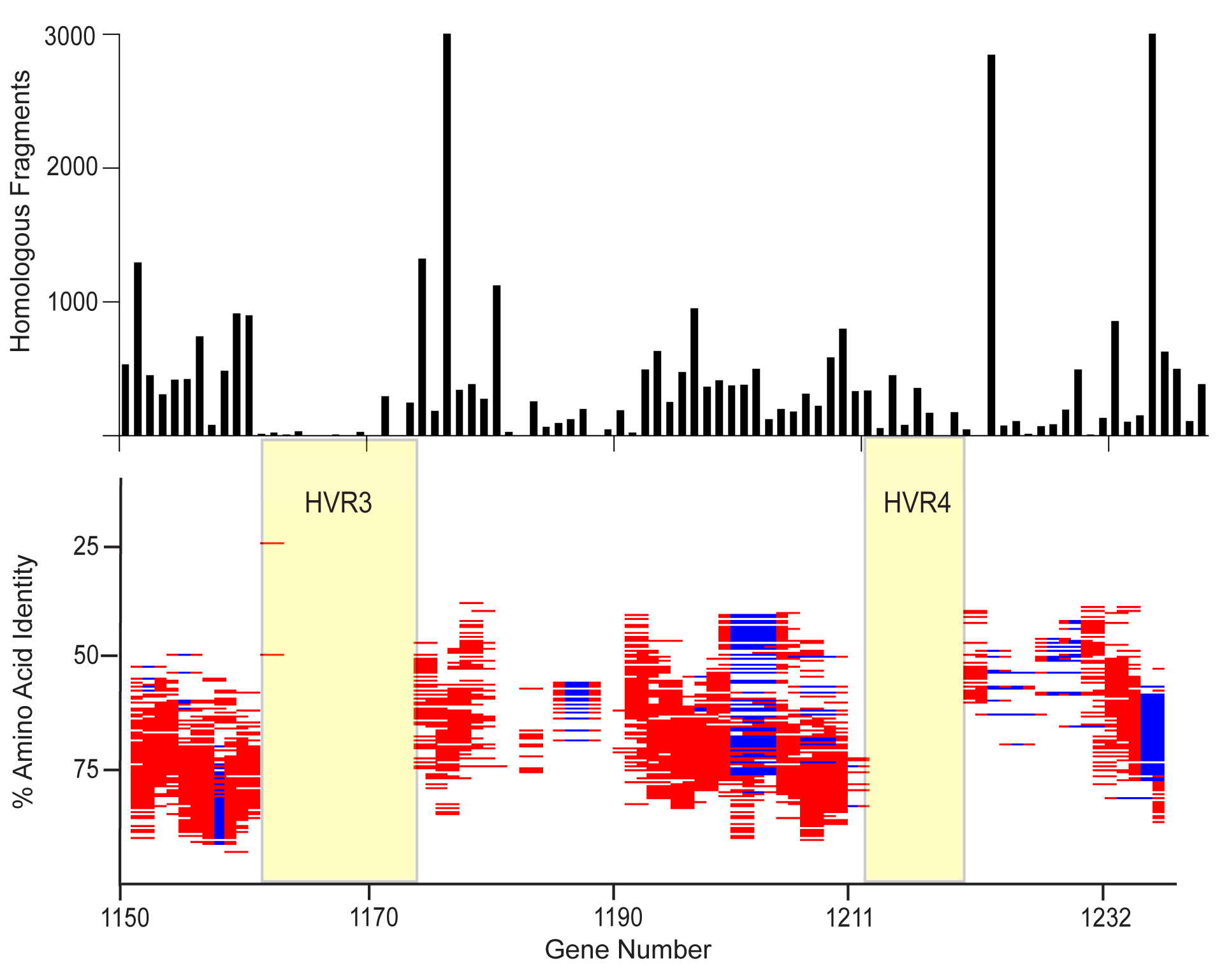

Supplement: Additional file 7 — Enlargement of HVR3 and HVR4. HTCC1062 syntenic fragment plot showing detail in the region of HVR3 and HVR4. [file 1745-6150-2-27-S7.doc]

**Enlargement of HVR1.**

**
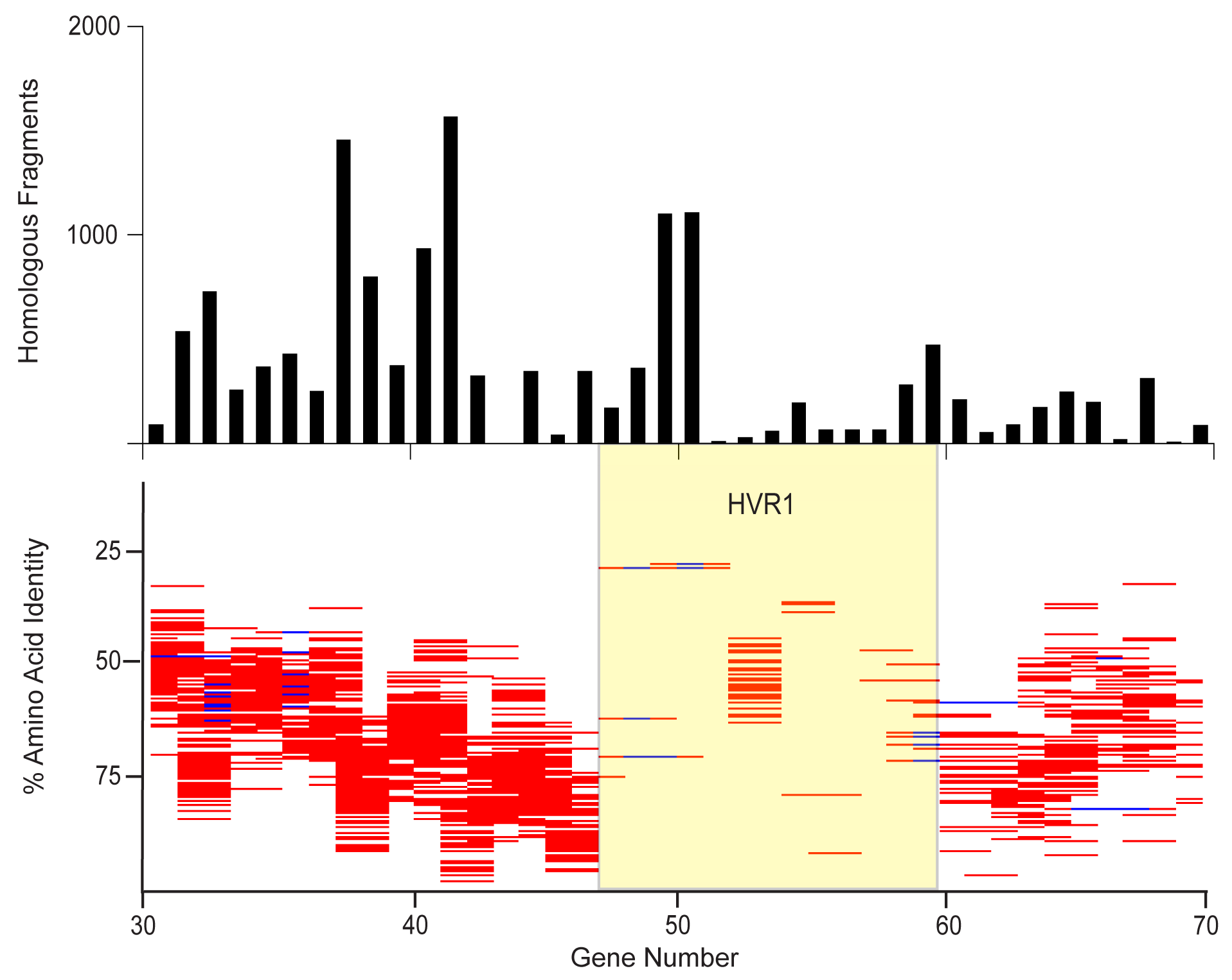
**

Supplement: Additional file 8 — Enlargement of HVR1. HTCC1062 syntenic fragment plot showing detail in the region of HVR1. [file 1745-6150-2-27-S8.doc]
